# A crisis in the shadows: public health outcomes and barriers to care for children of North Korean defectors in China

**DOI:** 10.3389/fpubh.2025.1652410

**Published:** 2025-10-14

**Authors:** Ji-Ung Jeong

**Affiliations:** Department of Disability Studies, Daegu University, Daegu, Republic of Korea

**Keywords:** healthcare access, health policy, statelessness, pediatric care, social determinants of health, North Korean defectors

## Abstract

**Background:**

Children born to North Korean defectors in China are effectively stateless and legally invisible, creating severe structural barriers to healthcare. This policy-driven public health crisis, marked by poor physical and mental health outcomes, remains critically underexamined from a healthcare perspective. This study aims to analyze the policy discourse to identify these structural barriers and understand how the health of this vulnerable pediatric population is framed and contested over time.

**Methods:**

A longitudinal qualitative content analysis was conducted on official documents from the UN Committee on the Rights of the Child (CRC) treaty-reporting cycles. The analysis covers the period from 2005 to 2022, systematically comparing the evolving positions of the Chinese government, the CRC, and civil society organizations on health-related issues.

**Results:**

The analysis reveals a persistent discursive stalemate. China consistently employs a strategy of deflection and denial, framing North Koreans as “illegal economic migrants” and a security threat, a position that has not changed over the 17-year period. In contrast, the CRC’s critique evolved from general concerns about “irreparable harm” to a specific focus on the *de facto* statelessness and denial of legal identity as the primary barrier to pediatric care for these children.

**Conclusion:**

State policies that deny legal identity are the primary structural determinants of this public health crisis, systematically producing poor health outcomes. The analysis confirms that while the UN reporting system is limited in its enforcement capacity, it is crucial for documenting these violations. To mitigate harm, future health policy and advocacy should focus on the key recommendation of decoupling access to essential pediatric services from a child’s legal or household registration status.

## 1 Introduction

The principle of universal access to healthcare is a cornerstone of global public health, predicated on the belief that a child’s health and survival should not be determined by their legal status (1, 2). However, state policies that render specific populations legally invisible create profound public health crises by systematically dismantling this access. This is the stark reality for an estimated 30,000–50,000 children of North Korean defectors in China, a hidden pediatric population facing extreme health risks due to their stateless and undocumented status (3, 4). Denied a legal identity, these children are systematically excluded from China’s public healthcare system, resulting in a cascade of negative health outcomes, including untreated pediatric illness, chronic malnutrition, and severe developmental trauma (5).

This public health crisis is not accidental but is engineered by specific state policies that function as powerful social determinants of health. The primary structural barrier is China’s household registration (*hukou*) system, which serves as an essential, non-clinical gatekeeper to all public services. As their North Korean mothers are undocumented and live under the constant threat of forced repatriation, the act of registering a child to secure healthcare access becomes a perilous choice that risks family separation and the mother’s return to a country where she faces torture or execution (5). This impossible dilemma effectively weaponizes the healthcare and administrative systems against them. Consequently, these children are barred from receiving essential pediatric care, including vaccinations and developmental screenings, while the perpetual fear of discovery acts as a source of chronic toxic stress—a well-documented physiological process that impairs childhood neurological development and precipitates significant mental health challenges in both mother and child (6–8).

While the devastating health consequences of this situation are known to humanitarian actors, they have been consistently marginalized within mainstream public health literature and policy discourse. The primary venue where this crisis is officially and publicly addressed is the UN human rights treaty-reporting system. Within this forum, bodies like the UN Committee on the Rights of the Child (CRC) have repeatedly challenged China on the physical and psychological harm caused by its policies (9, 10). This official dialogue, therefore, represents a unique site of analysis for understanding how the health of a vulnerable population is framed, contested, and ultimately neglected at the highest levels of international governance. This study repositions a human rights issue as an urgent public health problem. Through a longitudinal analysis of UN treaty body reporting documents from 2005 to 2022, this research dissects the political and legal architecture of this health crisis. The objective is to analyze how the health and wellbeing of these children are framed, how state justifications perpetuate barriers to care, and how the international community has responded. By moving beyond a description of the problem to an analysis of the policy discourse that sustains it, this study provides a new framework for understanding state-produced health inequities affecting hidden populations and offers critical insights for developing interventions that can mitigate harm and promote health justice.

This study uses three interconnected concepts to describe the plight of the children of North Korean defectors in China. ‘Lack of legal identity’ refers to the absence of official documentation which is the necessary prerequisite to access state services in China. This practical, bureaucratic exclusion often leads to ‘de facto statelessness,’ a condition where a child, despite potentially having a nationality in theory, is not recognized as a national by any state in practice and is thus denied the protections associated with citizenship. Together, these conditions create a state of ‘legal invisibility,’ a term used in this paper to describe the lived reality of being functionally non-existent to the state’s administrative, healthcare, and educational systems.

## 2 Literature review: a hidden public health crisis

This chapter establishes the context for the public health crisis facing children of North Korean defectors in China. It begins by describing the population and the environment of legal precarity in which they exist. It then details the specific physical and mental health vulnerabilities that define this pediatric crisis. Finally, it analyzes the state policies and legal frameworks that function as structural barriers to care, creating and perpetuating these adverse health outcomes.

### 2.1 Population of North Korean defectors and their children in China

Estimating the number of North Korean defectors residing in China is notoriously difficult, with figures ranging from tens of thousands to over 100,000 (11). This population exists in a state of extreme legal precarity, as China does not recognize them as refugees, but rather as illegal economic migrants (12). This classification leaves them without legal protection and under constant threat of discovery and forced repatriation to North Korea, where they face severe penalties, including torture and execution (11, 13).

Within this hidden population is a distinct and highly vulnerable pediatric group: children born in China to North Korean mothers and, typically, Chinese fathers. These children inherit their mothers’ precarious legal status. Their existence is defined by a lack of legal identity, as their mothers cannot safely register their birth without risking their own arrest (14). This legal invisibility is the foundational social determinant that drives the public health crisis affecting this group.

### 2.2 Physical and mental health outcomes of legal invisibility

The denial of legal status creates a cascade of severe and interconnected health crises for these children, impacting both their physical and mental wellbeing from birth. This section details the specific health profile of this hidden pediatric population.

#### 2.2.1 Physical health and barriers to pediatric care

The most immediate and damaging consequence of legal invisibility is the systemic exclusion from China’s public healthcare system. Lacking *hukou*, the essential household registration, these children are barred from receiving fundamental pediatric care, including the full schedule of routine childhood vaccinations, which places them at high risk for preventable infectious diseases such as measles, polio, and diphtheria (14). They are also excluded from developmental screenings, meaning that physical, cognitive, or sensory impairments (e.g., vision or hearing problems) may go undiagnosed during critical early-childhood windows, leading to lifelong disabilities that could have been mitigated with early intervention.

Furthermore, many of these children suffer from chronic nutritional deficiencies and stunted growth. This often begins *in utero*, as their mothers—frequently victims of trafficking and living in poverty—may have been malnourished during pregnancy, leading to low birth weight and a predisposition to poor health (15). This intergenerational transfer of vulnerability is compounded by post-natal poverty and food insecurity. Without access to subsidized healthcare or nutritional support programs, treating even common illnesses like respiratory infections or diarrhea becomes a significant financial burden. Families are forced to rely on costly private clinics they can ill afford or, more often, forgo care entirely. This not only leads to higher rates of preventable morbidity but also sets the stage for poor long-term health outcomes, as childhood malnutrition and stunting are linked to an increased risk of chronic diseases such as diabetes and cardiovascular conditions in adulthood (15).

#### 2.2.2 Mental health and developmental trauma

The environment of constant fear, instability, and secrecy inflicts profound and lasting psychological distress on these children. The perpetual threat of a mother’s arrest and forced repatriation creates a state of toxic stress, where the child’s developing brain is continuously exposed to high levels of stress hormones like cortisol. This is known to impair cognitive function, emotional regulation, and executive functioning (6). The common occurrence of family separation—whether through a mother’s arrest or her decision to flee onward to a third country—is a defining traumatic event. It can result in complex trauma (C-PTSD) and severe attachment disorders, as the child experiences a profound sense of abandonment and what trauma specialists term ‘ambiguous loss’: a psychologically devastating loss that lacks closure because the parent’s fate—whether they have been repatriated, imprisoned, or have fled elsewhere—remains unknown (6, 7, 16).

This direct psychological burden is compounded by severe social isolation. Taught from a young age to be inconspicuous and to avoid authorities, these children are often deprived of the normal peer interaction, play, and school environments that are crucial for healthy social and emotional development. This isolation can lead to feelings of otherness, loneliness, and depression, further hindering their ability to form healthy social relationships (8). The combination of these factors—toxic stress, trauma of separation, and social isolation—creates a mental health profile of extreme vulnerability, with children at high risk for a range of psychiatric and behavioral disorders that go entirely undiagnosed and untreated by any formal mental health service.

### 2.3 Structural determinants: state policies as barriers to healthcare

The adverse health outcomes detailed above are not accidental but are the predictable results of specific state policies that function as powerful structural determinants of health.

China’s policy of classifying North Korean defectors as “illegal economic migrants,” rather than as refugees or asylum seekers deserving of protection under international law, is the primary driver of this crisis (5, 13). This political and legal classification is a deliberate strategy that strips these individuals of any claim to international protection. The policy is enforced through a bilateral border agreement with North Korea and underpins the practice of forced repatriation. It is this credible and constant threat of repatriation that prevents mothers from seeking necessary prenatal and pediatric care for their children, effectively turning the healthcare system from a place of safety and support into a site of potential danger and family destruction.

Furthermore, China’s *hukou* system institutionalizes this exclusion, acting as the primary mechanism that translates a mother’s illegal status into her child’s denial of care. By linking access to all public services—including healthcare, vaccinations, and education—to this registration document, the system structurally denies care to any child who cannot be safely registered (14). Although China has ratified the Convention on the Rights of the Child, which mandates that the “best interests of the child” be a primary consideration in all actions, its domestic policies on immigration and household registration systematically override this principle for this specific population. These policies create a closed loop of vulnerability: the mother’s illegal status makes her child legally invisible, and this legal invisibility becomes an insurmountable barrier to accessing the healthcare necessary for a healthy and productive life.

## 3 Materials and methods

This chapter outlines the methodological approach used to analyze the policy discourse surrounding the health and wellbeing of children of North Korean defectors in China. It details the study’s health-focused research objectives, describes the data sources and selection criteria, and presents the systematic framework used for the qualitative content analysis.

### 3.1 Research objectives

The primary purpose of this study is to systematically analyze the international policy discourse that shapes the healthcare access and health outcomes of children of North Korean defectors in China. The research is guided by the central question: How has the policy dialogue within the UN system framed the health-related vulnerabilities and structural barriers to care for these children since 2000?

To understand the drivers of this public health crisis and inform future health policy, this research first identifies and categorizes the key health-related issues and barriers to care—such as the lack of access to pediatric services, mental health trauma, and nutritional deficits—as they are articulated within the UN CRC reporting process. The study then proceeds to analyze and compare how different actors, namely the Chinese government, the CRC, and civil society organizations, frame the health and wellbeing of these children. Finally, the research examines the evolution of this health policy discourse over time, assessing whether the focus on specific health outcomes has changed and how the Chinese state has responded to health-related recommendations.

### 3.2 Data sources

The primary data for this study consist of public documents from the CRC treaty-reporting system pertaining to the People’s Republic of China for reporting cycles after the year 2000. These legal and policy documents were selected as they represent the official, high-level discourse that directly influences the structural determinants of health for this population. The documents include State Party Reports submitted by China, Lists of Issues and Concluding Observations from the Committee, and Parallel Reports from civil society organizations.

Data collection was conducted using the UN Treaty Body Database. The search parameters were set to “Asia-Pacific - China” and “Committee on the Rights of the Child,” and relevant documents were downloaded. The specific documents selected for analysis are detailed in Table 1.

**Table 1:** Documents selected for analysis from the CRC treaty-reporting system.

Document classification	Producing organization		Nature of organization	Publication date
Concluding Observations (on 1st and 2nd State Report)	Committee on the Rights of the Child	UN	CRC treaty body	Nov 24, 2005
State Report (3rd and 4th periodic reports)	People’s Republic of China	China	State Party	Jul 16, 2010
Parallel Report	Human Rights in China (HRIC)	U. S.	Civil Society Organization	Nov, 2012
List of Issues	Committee on the Rights of the Child	UN	CRC treaty body	May 10, 2013
Reply to List of Issues	People’s Republic of China	China	State Party	Aug 29, 2013
Concluding Observations (on 3rd and 4th State Report)	Committee on the Rights of the Child	UN	CRC treaty body	Oct 29, 2013
State Report (5th and 6th periodic reports)	People’s Republic of China	China	State Party	Jul 16, 2022

This study employs a qualitative content analysis of public documents, a method well-suited for systematically identifying and interpreting specific themes and arguments within policy texts (17). The analytical procedure began with a thorough document immersion and open-coding process, where all text segments referencing North Korean defectors and their children were tagged with a specific focus on terms related to both legal status (e.g., refugee, statelessness) and health and wellbeing (e.g., healthcare access, mental health, nutrition). Following this coding, the initial codes were systematically reviewed and organized into broader, health-oriented themes, such as “Structural Barriers to Healthcare Access,” “Mental Health Stressors and Trauma,” and “State Justifications for Policies with Negative Health Impacts.” Next, the analysis proceeded with an actor-based comparative approach, where the content related to each theme was examined by its source—the Chinese government, the CRC, or civil society—to reveal how each actor framed the health issues. Finally, a longitudinal analysis was conducted across the reporting cycles (2005–2022) to trace the evolution of the health discourse, identifying persistent health concerns and any discernible shifts in the policy dialogue over time (Table 2).

**Table 2:** Analytical framework for the analysis.

Step	Title	Description
1	Document Immersion and Coding	Comprehensive reading and open-coding of documents to identify references to North Korean children related issues.
2	Thematic Categorization	Grouping codes into key themes.
3	Actor-Based Comparison	Analyzing each theme by source to identify consensus and divergence.
4	Longitudinal Analysis	Tracing thematic developments across reporting cycles to assess continuity and change over time.

## 4 Results

The longitudinal analysis of the UN treaty-reporting documents reveals a persistent and unresolved public health crisis affecting children of North Korean defectors in China. The dialogue between the CRC, the Chinese government, and civil society highlights several key themes: the profound health risks associated with China’s *refoulement* policy, the systemic denial of healthcare due to legal invisibility, and the discursive strategies used by the state that perpetuate these conditions.

### 4.1 The policy of forced repatriation: the risk of irreparable harm

The foundational issue that emerges from the UN reporting cycle is China’s policy of forced repatriation and its direct, severe health consequences. The dialogue began in 2005 when the CRC first expressed grave concern that children entering China from the DPRK were “categorically considered as economic migrants and returned… without consideration of whether there are risks of irreparable harm to the child upon return” (18). By invoking the term “irreparable harm,” the Committee framed the issue not merely as a legal violation but as a primary health concern, encompassing the immediate risks of physical violence and torture upon repatriation, as well as the long-term consequences of severe psychological trauma, such as Post-Traumatic Stress Disorder (PTSD) and chronic anxiety disorders.

In its 2010 State Report, the Chinese government responded not by addressing the risk of harm, but by employing a strategy of deflection. It highlighted its proper treatment of other, formally recognized refugee groups from countries like Vietnam, Pakistan, and Iraq, thereby projecting an image of compliance with international norms while sidestepping the specific allegations concerning North Koreans. The report firmly established a narrative that would persist for over a decade, stating that individuals from the DPRK are “not refugees” but illegal economic entrants, a classification that conveniently removes them from the protective obligations of the Refugee Convention (19).

This categorical denial was challenged by civil society, which pushed for a focus on procedure and individual health risks. In its 2012 submission, Human Rights in China (HRIC) argued that “the determination of refugee status and the risk of irreparable harm… is a factual, legal and individual determination,” urging the Committee to demand information on the specific procedural safeguards in place (20). Adopting this more pointed line of inquiry, the CRC’s 2013 List of Issues explicitly asked China to detail its “policies and mechanisms established… for assessing risk” (9). In its reply, the Chinese government escalated its rhetoric. It not only repeated its “illegal entrants” position but also actively securitized the population, framing them as a threat who “disrupted China’s normal order of entry and exit” and engaged in “criminal activities” (21).

This securitization of the defector population has direct and severe implications for public health. By labeling these individuals as criminals rather than a vulnerable population with health needs, the state narrative creates a powerful chilling effect, actively discouraging families from seeking any form of healthcare for fear that interacting with state authorities will lead to identification and repatriation. This fear becomes a primary structural barrier to accessing care. Furthermore, the ever-present threat of forced return functions as a source of chronic toxic stress, a well-established precursor to a range of adverse long-term physical and mental health conditions in both children and their caregivers. Thus, the state’s political and security framing is not merely a diplomatic posture; it is a policy choice that directly creates and exacerbates a public health crisis.

### 4.2 Legal invisibility as a structural barrier to pediatric care and education

The analysis of the UN reporting documents reveals a crucial evolution in the international discourse, moving beyond the immediate threat of refoulement for entering children to the chronic public health crisis facing children born inside China. The Committee’s 2013 Concluding Observations marked a key turning point by explicitly identifying that “children whose mothers are from the Democratic People’s Republic of Korea lack legal identity and access to basic rights, particularly education, as they are not registered under the hukou system” (10). The report further noted the “absence of special reception procedures… (and that they) lack access to health care” (10). This finding pinpoints the state-managed system of legal invisibility as the primary structural barrier to pediatric care.

At the heart of this barrier is China’s household registration system, or hukou. Far more than a census record, the hukou functions as an essential gatekeeper to all public services. For the undocumented North Korean mother, this creates an impossible choice: to secure her child’s legal right to healthcare by registering their birth, she must present her own identification, which would immediately expose her to arrest and forced repatriation. This catch-22 effectively weaponizes the healthcare and administrative systems against her, turning a potential act of care into one of extreme personal risk. Consequently, a rational fear for her own life and of permanent separation from her child forces her to keep the child unregistered and, therefore, outside the formal healthcare system.

The health consequences of this systemic exclusion are severe and multifaceted. The lack of legal identity systematically denies these children access to fundamental preventative health measures, such as routine childhood immunizations, which leaves both the individual child and the wider community vulnerable to outbreaks of preventable diseases. They are also excluded from developmental screenings, meaning that physical or cognitive delays may go undiagnosed and untreated during critical developmental windows. Access to primary care for acute illnesses is limited to costly private clinics or is forgone entirely, increasing the risk of complications and preventable morbidity.

Furthermore, the Committee’s explicit concern about access to education is also a critical public health issue. Education is a well-established social determinant of long-term health and wellbeing, strongly correlated with health literacy, lifestyle choices, and lifetime economic stability (22). By being barred from the state school system, these children are placed on a trajectory of poor long-term health outcomes. In its official reports, including the most recent one from 2022, the Chinese government remains completely silent on the specific issue of birth registration for this population, thereby reinforcing their invisibility at a policy level and ensuring that the structural barriers to healthcare and its social determinants remain firmly intact (23).

### 4.3 State justifications and the persistence of the public health crisis

Across the entire 17-year period of analysis, a clear and unchanging pattern of discursive stalemate emerges. This stalemate is not passive; it is an active policy environment that directly explains the persistence of the public health crisis facing children of North Korean defectors. The pattern is consistently initiated by the CRC and civil society raising specific, rights-based health and welfare concerns, which are then met with a robust and unvarying three-part defensive strategy from the Chinese government: deflection, denial, and securitization.

The first element of this strategy is deflection. China repeatedly points to its positive treatment of recognized refugee populations from other countries, such as those from Indochina, Pakistan, and Iraq, to project an image of compliance with international norms (19, 21). This rhetorical move is a well-documented state practice of “organized hypocrisy,” where states publicly commit to international norms while selectively violating them to protect sovereign interests (24). This allows China to create a narrative of a functioning and humane refugee system, while simultaneously carving out a political exception for North Koreans, effectively insulating its policy toward them from the same standards.

This is immediately followed by denial, achieved through a powerful act of legal framing. The cornerstone of China’s position is its categorical denial that North Koreans qualify as refugees. By consistently labeling them as “illegal economic migrants,” the state transforms a humanitarian issue requiring protection into a simple matter of domestic immigration law enforcement (19, 21, 22). This legal re-framing has profound health implications: it absolves the state of any obligation to provide specialized care or protection under international refugee law and justifies its primary policy response of repatriation.

Finally, the strategy is reinforced by securitization. This is a process where an issue is framed as an existential threat to the state, thereby justifying extraordinary measures outside the realm of normal politics (25). In its 2013 reply, the Chinese government escalated its rhetoric by framing defectors as a threat to public order who “disrupted China’s normal order of entry and exit” and engaged in “criminal activities” (21). This move reframes the population from a vulnerable group with health needs to a security threat to be managed. This has a direct chilling effect on health-seeking behaviors, a phenomenon well-documented among undocumented populations who fear that interacting with any state institutions—including hospitals and clinics—will lead to identification and deportation (26). The state’s political and security framing, therefore, functions as a direct structural barrier to health.

The persistence of this stalemate demonstrates that the policy environment itself perpetuates poor health outcomes. China’s 2022 report shows this position has not evolved, repeating the same justifications from previous cycles (22). This refusal to engage with the health and welfare concerns raised by the Committee creates a form of structural violence, where the political and legal systems systematically inflict harm and prevent a vulnerable population from meeting its basic health needs (27). The result is not merely a diplomatic impasse but a sustained policy environment that knowingly creates and perpetuates a public health crisis.

## 5 Conclusion

This study investigated the public health crisis facing children of North Korean defectors in China—an acutely vulnerable group rendered stateless and invisible by structural policy exclusions. A longitudinal analysis highlights a persistent discursive stalemate, driven by a fundamental clash between China’s state-centric security framework and the UN’s rights-based public health principles. As a result, these children remain largely excluded from pediatric care and public health services. As a result, these children remain largely excluded from pediatric care and public health services.

The analysis traced the evolution of the UN CRC’s concerns—from the risk of irreparable harm through forced repatriation to the entrenched crisis of *de facto* statelessness among these children. Because they are systematically denied legal identity under the *hukou* system, these children lack access to essential health services such as immunizations, growth monitoring, and mental health support. China’s continued refusal to engage substantively with these critiques ensures that its policy regime not only perpetuates but structurally produces poor health outcomes. The policy environment itself, shaped by securitization and sovereign control, becomes a determinant of health inequality.

The structural mechanisms identified in this study—where legal status is used as a gatekeeper to healthcare—are not unique to China but reflect a global pattern of state-produced health inequity. The absolute exclusion and dire health outcomes faced by Rohingya children in Bangladesh, who are also denied citizenship and legal identity, offer a stark parallel, particularly concerning malnutrition and vulnerability to infectious diseases (28). Similarly, the “chilling effect” of state policy is observed in Western nations, where the fear of immigration enforcement deters undocumented families in the United States and Europe from accessing necessary pediatric care (29). While the specific policy tools differ, the outcome is consistent: the state’s control over legal status becomes a powerful mechanism for producing health inequities among vulnerable children.

This case reflects broader insights from global health and political theory. It exemplifies how state policy can function as a powerful social determinant of health—where decisions about legal recognition and administrative classification carry direct consequences for physical survival (30). China’s approach, rooted in a biopolitical logic of population control and selective inclusion, places children born to undocumented migrants outside the boundaries of public care. It also creates an ethical bind for health professionals, who may hesitate to offer care for fear of triggering punitive state responses toward their patients and their families (31). This dynamic aligns with what some scholars have termed “letting die” as an exercise of sovereign power (32).

Addressing this crisis in a realistic and constructive way requires a public health strategy that acknowledges China’s political constraints while seeking feasible pathways to mitigate harm. Rather than appealing solely to refugee law or moral imperatives international advocacy should emphasize low-profile, rights-compatible policies already recognized in principle by China. For example, targeted support for local-level pilot programs that provide limited healthcare access to undocumented children under the umbrella of infectious disease control or maternal and child health could be framed as contributing to national public health goals. Quiet engagement with municipal governments in border regions or urban areas with known defector populations may yield more cooperation than direct political confrontation.

Community-based NGOs and religious organizations operating in China or neighboring countries may also play a role by discreetly supporting informal health education, mobile clinics, and nutritional aid without drawing overt attention to the legal status of the recipients. International organizations could explore partnerships with these actors to extend existing immunization programs to children in undocumented households, in ways that minimize state scrutiny.

Finally, there is a pressing need for empirical public health research that documents the health needs and risks faced by these children. Epidemiological and qualitative studies conducted with resettled defector families in South Korea or elsewhere could provide the data necessary to inform harm-reduction strategies and identify indirect indicators of the crisis in China. These findings can support evidence-based policy recommendations that align with China’s stated commitments to children’s health, without requiring a fundamental shift in political ideology.

This study is limited by its reliance on official UN documents which may not fully capture the lived experiences of children and families affected by structural exclusion. Specifically, the use of treaty-body reports introduces potential bias, as these documents reflect formal state responses and international advocacy perspectives rather than direct ethnographic or testimonial evidence. As a result, gaps likely remain regarding local practices and informal health access. This methodological limitation suggests that the findings may be conservative; the true extent of exclusion and associated health risks may be even more severe than indicated. Nevertheless, by foregrounding the intersection of legal status and public health, it contributes to a clearer understanding of how structural exclusion produces preventable suffering. Making these children visible in international and domestic health policy discourse is an urgent step—not only toward protecting their right to health but also toward restoring their basic human dignity in a context that has long denied it.

## Data Availability

All primary source documents cited and analyzed in this study are open access and can be accessed directly through the United Nations Treaty Body Database.

## References

[1] Palm W Hernandez-Quevedo C Klasa K van Ginneken E. Implementation of the right to health care under the UN convention on the rights of the child. Copenhagen: European Observatory on Health Systems and Policies (2017).

[2] Onarheim KH Melberg A Meier BM Miljeteig I. Towards universal health coverage: including undocumented migrants. BMJ Glob Health. (2018) 3:e001031. doi: 10.1136/bmjgh-2018-001031, PMID: 30364297 PMC6195153

[3] Kim SW. North Korean refugees and China’s responsibility. New Asia. (2012) 19:55–83.

[4] The Guardian. 30,000 North Korean children living in limbo in China. (2016). Available online at: https://www.theguardian.com/world/2016/feb/05/north-koreas-stateless-children (Accessed October 16, 2023).

[5] Son HJ. A study on the legal status of North Korean defectors. Leg Stud. (2017) 53:109–47. doi: 10.22851/kjlr.2017..53.003

[6] Baik J Yoon YJ Gibson P Lo N Nam HE Im YJ. Mothering and mothered during defection and resettlement: experiences of North Korean refugee women and their children. Child Youth Serv Rev. (2021) 130:106242. doi: 10.1016/j.childyouth.2021.106242

[7] Lee K Kim MK Baik JW. A study on the child-raising experiences of North Korean defector women with children born in China. J Korean Fam Relat Assoc. (2014) 19:213–40.

[8] Stodolska M. Leisure during the escape and adaptation in South Korea: a life story of a North Korean adolescent defector. Leis Sci. (2024):1–20. doi: 10.1080/01490400.2024.2348500

[9] Committee on the Rights of the Child. List of issues in relation to the combined third and fourth periodic reports of China. Geneva: United Nations (2013).

[10] Committee on the Rights of the Child. Concluding observations on the combined third and fourth periodic reports of China, including Hong Kong, China and Macao, China. Geneva: United Nations (2013) Report No.: CRC/C/CHN/CO/3-4.

[11] Chong S Choi H. A study on the legal status and protection of North Korean defectors in China: Focusing on the comparison with Chinese legal system & international refugee law. Public Law Study. (2017) 8:111–141. doi: 10.31779/plj.18.4.201711.005

[12] Kang C. The aquariums of Pyongyang: ten years in the North Korean gulag. New York (NY): Basic Books (2003). 170 p.

[13] Han HW. A normative study on the right enactment direction of the North Korean human right act under the new circumstance and the legality of north Korean defectors under international refugee law. J Leg Stud. (2013) 50:29–53.

[14] McPhee S. Kotjebi: North Korean children in China. Asian Aff. (2014) 45:484–9. doi: 10.1080/03068374.2014.951564

[15] Choi SW. Changes in health status of North Korean children and emerging health challenges of north Korean refugee children. Clin Exp Pediatr. (2021) 64:552–8. doi: 10.3345/cep.2021.00192, PMID: 34015896 PMC8566798

[16] Boss P. Ambiguous loss: working with families of the missing. Fam Process. (2002) 41:14–7. doi: 10.1111/j.1545-5300.2002.40102000014.x, PMID: 11924081

[17] Pinto AD Manson H Pauly B Thanos J Parks A Cox A. Equity in public health standards: a qualitative document analysis of policies from two Canadian provinces. Int J Equity Health. (2012) 11:21. doi: 10.1186/1475-9276-11-28 22632097 PMC3479047

[18] Committee on the Rights of the Child. Concluding observations: China. Geneva: United Nations (2005) Report No.: CRC/C/CHN/CO/2.

[19] People’s Republic of China. Third and fourth combined report on the implementation of the convention on the rights of the child. Geneva: United Nations (2010).

[20] Human Rights in China. Suggested questions and issues to be raised with the Chinese government in advance of the review of its third report on the implementation of the convention of the rights of the child. New York (NY): Human Rights in China (2012).

[21] People’s Republic of China. Response of the Chinese government to questions concerning the combined 3rd and 4th periodic reports on the implementation of the convention on the rights of the child. Geneva: United Nations (2013).

[22] The Lancet Public Health. Education: a neglected social determinant of health. Lancet Public Health. (2020) 5:e361. doi: 10.1016/S2468-2667(20)30144-4 32619534 PMC7326385

[23] People’s Republic of China. Combined fifth and sixth reports on the implementation of the convention on the rights of the child. Geneva: United Nations (2022) Report No.: CRC/C/CHN/5-6.

[24] Krasner SD. Sovereignty: organized hypocrisy. Princeton (NJ): Princeton University Press (1999).

[25] Buzan B Wæver O de Wilde J. Security: a new framework for analysis. Boulder (CO): Lynne Rienner Publishers (1998).

[26] Friedman A Venkataramani AS. Chilling effects: US immigration enforcement and health care seeking among hispanic adults. Immigrant Health. (2021) 40:1056–65. doi: 10.1377/hlthaff.2020.02356 34228522

[27] Farmer PE. An anthropology of structural violence. Curr Anthropol. (2004) 45:305–25. doi: 10.1086/382250

[28] Islam MM Nuzhath T. Health risks of Rohingya refugee population in Bangladesh: a call for global attention. J Glob Health. (2018) 8:1–4. doi: 10.7189/jogh.08.020309 PMC6220352 30410735

[29] Corona Maioli S Bhabha J Wickramage K Wood LCN Erragne L Ortega García O. International migration of unaccompanied minors: trends, health risks, and legal protection. Lancet Child Adolesc Health. (2021) 5:882–95. doi: 10.1016/S2352-4642(21)00194-2, PMID: 34416189 PMC7615140

[30] Marmot M Allen J Bell R Bloomer E Goldblatt P Consortium for the European Review of Social Determinants of Health and the Health Divide. WHO European review of social determinants of health and the health divide. Lancet. (2012) 380:1011–29. doi: 10.1016/S0140-6736(12)61228-8 22964159

[31] Foucault M. The birth of biopolitics: lectures at the Collège de France, 1978–1979. New York (NY): Palgrave Macmillan (2008).

[32] Willen SS Knipper M Abadía-Barrero CE Davidovitch N. Syndemic vulnerability and the right to health. Lancet. (2017) 389:964–77. doi: 10.1016/S0140-6736(17)30261-1, PMID: 28271847

